# Introductory Lecture before the Class in the Ohio Dental College, Oct. 15, 1874

**Published:** 1875-01

**Authors:** J. S. Cassiday

**Affiliations:** Professor of Chemistry


### INTRODUCTORY LECTURE BEFORE THE CLASS IN THE OHIO DENTAL COLLEGE, OCT. 15, 1874.

BY J. S. CASSIDAY, D. D. S., PROFESSOR OF CHEMISTRY.

*Gentlemen:*—I do not think it altogether the proper thing for me to impose on you a lesson on this the occasion of our first meeting, but as preliminary to our regular course of lectures, I propose to occupy a portion of our hour to-day with a few informal remarks on the status of chemical knowledge in the past.

In order to appreciate the history of chemistry *as a science*,* we do not have to seek amid the dim, almost unlettered pages of the distant past.

The first authentic evidenee of investigations into the nature of matter, appears to the credit of the Egyptians; and one of them, named Trismegistus, is represented as the father of alchemy. He labored, as all subsequent alchemists did, in the vain effort of discovering a process for converting baser substances into gold and silver, and of eliminating from some mysterious mixture, the elixir of life, the liquid that would, by a simple taste, restore to the aged, tottering, weak, and withered man, his lost youth, strength, elasticity, and beauty. Such was the object of what is known as alchemy—and the most we can say of its followers derogatory to their honored memory—is that they were alas! victims of misplaced confidence.

The alchemy of the Arabs came later, and was far more practical and useful than that of the Egyptians. *Their* dream also was Transmutation, but in their blind gropings after the impossible they discovered many valuable compounds and methods of investigation. They had their pestles and mortars, crucibles and furnaces, alembics and aludels; they had

* The ancients were, no doubt, familiar with a vast amount of useful facts pertaining to the domain of material nature, but those facts were a mere heterogeneous collection, compared to the definitely arranged scientific truths of to-day.

vessels for infusion, decoction, sublimation, filtration, coagulation, etc. They worked with gold, silver, mercury, arsenic and sulphur; salts and acids, and indeed became acquainted with a large number of bodies, now called chemicals. They prepared salammoniac by heating camel's dung. They taught that there were but three elemental substances, mercury, sulphur and arsenic, and these, especially the first two, seemed to attract their exclusive and enthusiastic attention. They considered the metals as compounds made up of mercury and sulphur, united in different proportions. They saw mercury dissolve gold—the most incorruptible of bodies, as water dissolves sugar, and a piece of sulphur presented to red hot iron, penetrates it like a spirit and makes it run down like a shower of solid drops, a new and remarkable substance, possessed of properties belonging to neither iron or sulphur. One of their number named Geber, wrote a book on the subject, and called it "The Summit of Perfection." It is the oldest known book on chemistry, and although full of ridiculous nonsense, it is truly a wonderful performance.

From the Arabs, alchemy found its way through Spain into Europe, and soon became entangled with the fantastic mysticisms of the scholastic philosophy then in existence. The earliest works on alchemy are those of Roger Bacon and Albertus Magnus. Both of whom flourished contemporaneously, in the thirteenth century. Bacon was acquainted with the properties of a mixture now known as gunpowder, and although he condemned magic, necromancy, charms and such things, he firmly believed in the convertibility of the inferior metals into gold. He had more faith however, in the so-called Elixir of Life, than in gold producing. He followed the Arabian, Geber, in regarding the solution of gold in aqua regia as the true elixir, and cites a supposed case of an old man, who, plowing one day in Italy, found some yellow liquid in a golden vial, and thinking it was dew, he drank it off, and was immediately transformed into a strong and highly accomplished youth.

Albertus Magnus, in addition to the mercury and sulphur theory of the metals of the Arabians, regarded water as much nearer the soul of nature than either of those bodies. He appears indeed to have thought it the primary source of all things.

Thomas Aquinas was the first to use the word amalgam, and Raymond Lully the first to introduce the use of chemical symbols, his system consisting of a scheme of arbitrary hieroglyphics. Basil Valentine was the first to use antimony as a medicine. He regarded mercury and sulphur and common salt, as the three bodies contained in the metals. He inferred that the philosopher's stone must be a compound of the same substances, so pure that its projection on the baser metals, should be able to work them up into a condition of greater and greater purity, bringing them at last, to the desired purity of silver and gold. His practical knowledge however, was very extended. He knew how to precipitate iron from solution by potash, and many other similar processes, so that he is regarded as the founder of analytical chemistry.

But perhaps the most famous of all the names in the annals of alchemy, is that of Paracelsus. He agreed with Valentine that the elements of compound bodies were salt, sulphur, and mercury. All kinds of matter were, he thought, reducible under one or other of these typical forms. Everything was either a salt, a sulphur, or a mercury, or, like the metals, a compound of these. There was one element, however, common to the four; a fifth essence, or quintessence of creation; an unknown and only true element of which the four generic principles, namely salt, sulphur, mercury and the metals, were nothing but derivative forms; in other words, he taught that there is only *one* real elementary matter—no body knows what. This one prime element of things, he considered to be the universal solvent, of which the alchemists were in search.

After Paracelsus, the alchemists of Europe, became divided into two classes; one class was composed of sensible men, practical workers and observers of facts. The other class took up the visionary fantastic side of the older alchemy and carried it to a degree of absurdity before unknown.

The seven metals correspond with the seven planets, the seven openings of the head, the eyes, the ears, the nostrils, and the mouth. Gold was Apollo, silver was Diana, iron was Mars, tin was Jupiter, lead was Saturn, and so on. They talk incessantly of the power of attraction, which drew all men and women after the fortunate possessor; (and let me here venture to remark in all seriousness that there is a considerable number of people in these enlightened states and cities of America who have a notion that there is a substance of some kind, which confers upon the possessor an irresistible power of attraction, and that the doctors know all about it). They talked of the *Alcahest*, the spirit of matter, and of the grand elixir which was to give an immortality of youth to the student, who should approve himself pure and bold enough to kiss and quaff the golden draught. There was the great "mystery" the mother of the elements and the grandmother of the stars. It was in connection with this mock alchemy, mixed up with astrology and magic, that barefaced quackery abounded, and indeed it is so to a not inconsiderable extent at the present time.

It is from this degenerate class of visionaries and imposters that the prevailing idea of alchemy is derived, an unjust aspersion upon the character of the really meritorious alchemists who paved the way for the advancement of genuine chemistry. Many of them were too intelligent and practical, to waste their time and substance in dreaming of the golden terrestrial immortality, to be found only in the mythical "Philosopher's Stone." Away back in the distant ages of Egyptian civilization, we find evidences of a most wonderful knowledge of chemical reactions and processes for preserving dead bodies from decay, which no modern chemistry could excel. They prepared medicines and pigments, metals and metallic alloys, soap and beer, and vinegar, and common salt, soda and sal ammoniac, glass, enamel, tiles and painted earthenware; and even the Chinese long ages ago were acquainted with the preparations of sulphur, nitre, borax, alum, verdigris; porcelain, paper and gunpowder. But they and their successors of all the nations groped in the midst of the tantalizing wealth of nature's laboratory during many hundred dreary years. The rest of them were merely artistic and scientific.

The first evidence of a real science of chemistry, appeared after the discovery by Brandt in the latter half of the seventeenth century, of the weirdlike luminous body called phosphorus. He had worked many weary days and nights, with the hope of obtaining, from disgustingly concentrated urine, a solvent which would change silver into gold, when to his astonishment he saw a residuum in his retorts, of a waxy nature, which burst into flame by subjection to the slightest warmth.

This discovery of a hitherto unknown element, although purely accidental and cherished as a costly secret for many years, appears to me to mark the beginning of modern chemistry, especially when taken in connection with the fact that soon afterward, Sylvius, and Thomas Willis, and Boerhaav established an important school of medicine founded mainly upon chemistry and mechanics.

To the more positive thinkers, however, as Lavoisier, Priestly, Schul, Galvani, Volta, Sir Humphrey Davy, Faraday, Berzelius and other names, of almost equal brilliancy, chemistry owes its rapid exaltation into a science.

It is claimed, perhaps justly too, that the discovery of oxygen by Priestly one hundred years ago, marks the birth of scientific chemistry, and therefore its centennial birth-day has been celebrated in different lands in this year of grace 1874, with a noble pride in the object and subject, and an enthusiastic fervor worthy of the cause.

I have, gentlemen, thus sketched, briefly as possible, an imperfect outline of the origin and progress of our favorite science. I have, for obvious reasons, purposely avoided any allusion to the various complicated theories promulgated at different times. And neither have I referred to the present status of chemistry for the good reason that it would be impossible to do justice to the immensity of the subject. It is indeed as illimitable as the universe itself, and he who would even approximate a mastery of its practical philosophy must spend in its study a lifetime of labor and love.

